# Multi-Morbidity at Death and the US Disadvantage in Mortality

**DOI:** 10.1007/s10680-025-09749-3

**Published:** 2025-10-23

**Authors:** Magali Barbieri, Aline Désesquelles, Viviana Egidi, Luisa Frova, Francesco Grippo, France Meslé, Marilena Pappagallo, Sergi Trias-Llimós

**Affiliations:** 1 Istituto Nazionale Di Statistica, Rome, Italy; 2 National Institute for Demographic Studies (INED), Paris, France; 3 University of California, Berkeley, USA; 4 Sapienza University of Rome, Rome, Italy; 5 Center for Demographic Studies (CED-CERCA), Barcelona, Spain

**Keywords:** Mortality, Multi-morbidity, Multiple causes of death, United States, Comparative analysis

## Abstract

The US experiences significant excess mortality compared to peer countries. The literature indicates that a similar disadvantage affects morbidity and, more generally, the prevalence of risk factors for major diseases within the US population. In this study, we assess the impact of multi-morbidity at death on the mortality gap between the US and three other high-income countries with comparable data, namely France, Italy, and Spain. The study relies on an analysis of the multiple cause-of-death information available on all death certificates for 2017, used to classify morbid processes leading to death into three categories: simple, multi-morbid, and ill-defined. The results show disproportionately high rates of multi-morbid processes in the US compared with the other three countries. Multi-morbid processes contribute 51% of the US gap in life expectancy at birth with Italy, 73% with Spain, and 75% with France, with a particular concentration at ages 20–85 years. The prevalence of multi-morbid processes in the US is consistent with the hypothesis that multiple factors, rather than a single culprit, are at play in the disadvantage in mortality and it could explain, at least in part, the extraordinarily high cost of health care in this country.

## Introduction

Progress in survival in the United States (US) has been lagging behind that in peer countries starting around 1980. The phenomenon has generated a large body of literature, including two comprehensive reports by the National Research Council (National Research Council, 2011 and 2013) and a more recent study by the National Academies of Sciences, Engineering, and Medicine (Harris et al., 2021). These and other works have highlighted the characteristics of the US shortfall in survival. They demonstrated that the US experiences higher mortality at every age, though which age group most contributes to the difference with peer countries depends on the indicators used for the comparison. While ages below 65 and, especially, below 50 years most contribute to the divergence in the age-specific mortality rates, older ages (65 years and above) contribute more to the growing gap in life expectancy at birth (Barbieri, 2021; Dowd et al., 2024; Ho, 2013; Ho & Preston, 2010; Manton & Vaupel, 1995). They further showed that the US disadvantage is attributable to all causes of death, with external causes (including drug overdoses) contributing more at younger adult ages and cardiovascular diseases at older ages (Timonin et al., 2024). Thus, no specific age group or disease has been identified as the single culprit for the US disadvantage in mortality, necessitating a global, rather than focused, examination to better understand the situation.

The literature suggests that rates of morbidity for most chronic diseases, major risk factors, and disability, are higher in the US than in most peer countries, with higher prevalences of cardiometabolic diseases, some cancers, digestive diseases, hypertension, diabetes, and obesity (Banks et al., 2006; Chowdhury et al., 2023; Garmany & Terzic, 2024; Ho et al., 2021; Martinson et al., 2022; Murray et al., 2013; Pongiglione et al., 2022; Salive, 2013). The prevalence of multi-morbidity appears to have increased for cohorts born since World War II in the US (Bishop, Haas and Quinones, 2022; Freedman et al., 2013; King et al., 2018; Martin & Schoeni, 2014). The phenomenon is not specific to the US but its acceleration has been faster there than in European countries (Gimeno et al., 2024; Haas et al., 2017; Lafortune & Balestat, 2007). Two factors operate in concert to increase the share of the population with multi-morbidities in low mortality countries, namely medical progress and population aging. Medical progress and the ability to prevent deaths from a range of chronic conditions extends the lives of people affected by these conditions. The situation is exasperated by population aging as older people are more likely to suffer from concomitant chronic diseases and conditions since time and the increased vulnerability with age are major factors in the accumulation of health problems.

In a context of rapidly aging societies and the rising burden of chronic diseases due to improved survival from cancer, cardiovascular diseases, and other conditions, monitoring the prevalence of multi-morbidity is essential for public health. Furthermore, the increasing burden of obesity, diabetes, and hypertension in most populations, together with the expected multiplication of health crises such as pandemics and extreme weather events due to globalization and climate change generate susceptibilities in the population that could further increase the prevalence of multi-morbidity, as exemplified by the prevalence of long COVID, a risk factor for death in people with pre-existing conditions (Frieden et al., 2021; Haileamlak, 2022; Lipski, 2024; Parohan et al., 2020; Zhang et al., 2023). Such crises are known to both weaken individuals affected by chronic diseases, thus precipitating death, and create new fragilities in previously healthy individuals (Ashad-Bishop et al., 2023; Del Rio et al., 2020; Higgins et al., 2021; Hilser et al., 2024; Wang et al., 2023).

It is unclear to which extent the US multi-morbid pattern affects its mortality disadvantage as few studies have reliably assessed the contribution of various morbid processes. However, determining whether some countries exhibit a relative advantage in the prevalence of multi-morbidity is an important step to identify possible mitigating factors and to develop strategies and programs to alleviate the burden of poor health in later life (Navickas et al., 2016). Those who have attempted to conduct comparative analyses all acknowledge major weaknesses in their work due to heterogeneity in the setting of the studies they use for their comparisons. They point to the lack of standard methods for measuring multi-morbidity, the diversity of data sources and population samples, differences in the timing of data collection and in the definition of basic concepts, including multi-morbidity, and variations in the specific diseases and conditions selected for study (Boeckxstaens & Petrovic, 2020; Chua et al., 2021; Ho et al., 2021, 2022; Ricci-Cabello et al., 2015; Wittenberg, 2015).

Our goal is to fill some of the gap in knowledge about the contribution of multi-morbidity to the US disadvantage in mortality by examining the issue from a different angle than has been the case so far. Instead of combining different sources of data on co-morbidities in living populations, we are leveraging routinely collected data on causes of death to look at differences in the prevalence and pattern of multi-morbidity at death in the US and three high-income countries with very low mortality, namely France, Italy and Spain. As further explained below, we measure multi-morbidity strictly from the information available on death certificates. We use these data to compute a range of mortality indicators, each providing a different perspective on mortality patterns. We first compute age-specific and age-standardized mortality rates; we then assess the contribution of mortality in each age group to the US shortfall in life expectancy; and we calculate the number of excess deaths attributable to each process and the years of life that have been lost due to the US higher level of mortality. These calculations are carried out for each type of morbid process.

## Data and method

The comparison countries have been chosen based on their low levels of mortality, the availability and accessibility of the complete multiple cause-of-death data, the comparability of their data collection and processing systems, similarly aging populations, and high-quality health care systems. The selected set includes France, Italy, and Spain, which rank among the top countries in the world in terms of life expectancy at birth. Like the US, the three countries are high-income democracies and all four study countries are large enough for statistical analysis. The study relies on the processing of the multiple cause-of-death information coded to the 10th Revision of the International Classification of Diseases (ICD-10) available in the national vital statistics systems of each of the four countries: the National Institute for Health and Medical Research (INSERM) in France, the National Institute of Statistics (ISTAT) in Italy, the National Institute of Statistics (INE) in Spain, and the National Center for Health Statistics (NCHS) at the Centers for Disease Control (CDC) in the US. All four countries implement automatic coding of the death certificates using similar software. We focused on the most recent year (2017) for which data were available in all four countries at the time of this analysis. The total number of deaths reached 424,523 in Spain, 591,530 in France, 650,590 in Italy, and 2,813,374 in the US.^1^ Death records were available by sex, age, and for all the medical causes reported on the certificate.

The four countries follow the guidelines of the World Health Organization (WHO, 2016) and use the recommended death certificate on which reporting physicians, medical examiners, or coroners list all the causes which have directly or indirectly contributed to death. Causes of death are listed on two different sections of the standard certificate form. Part I records the sequence of diseases or conditions which have ended in death, i.e., all medical causes involved in "the sequence of morbid conditions, lesions or poisoning that led directly to death" (WHO, 2015). This section theoretically includes the "underlying cause of death", that is the disease or injury that initiated the chain of events leading to death. Part 2 records all other causes which were not directly responsible for the sequence listed in Part 1 but which nonetheless "contributed significantly to death" (ibid.) by influencing the course of the morbid process, often designated as the associated or contributing causes. The analysis presented here relies on these multiple causes of death, which include all the causes listed anywhere on the death certificate.

**Fig. 1:**
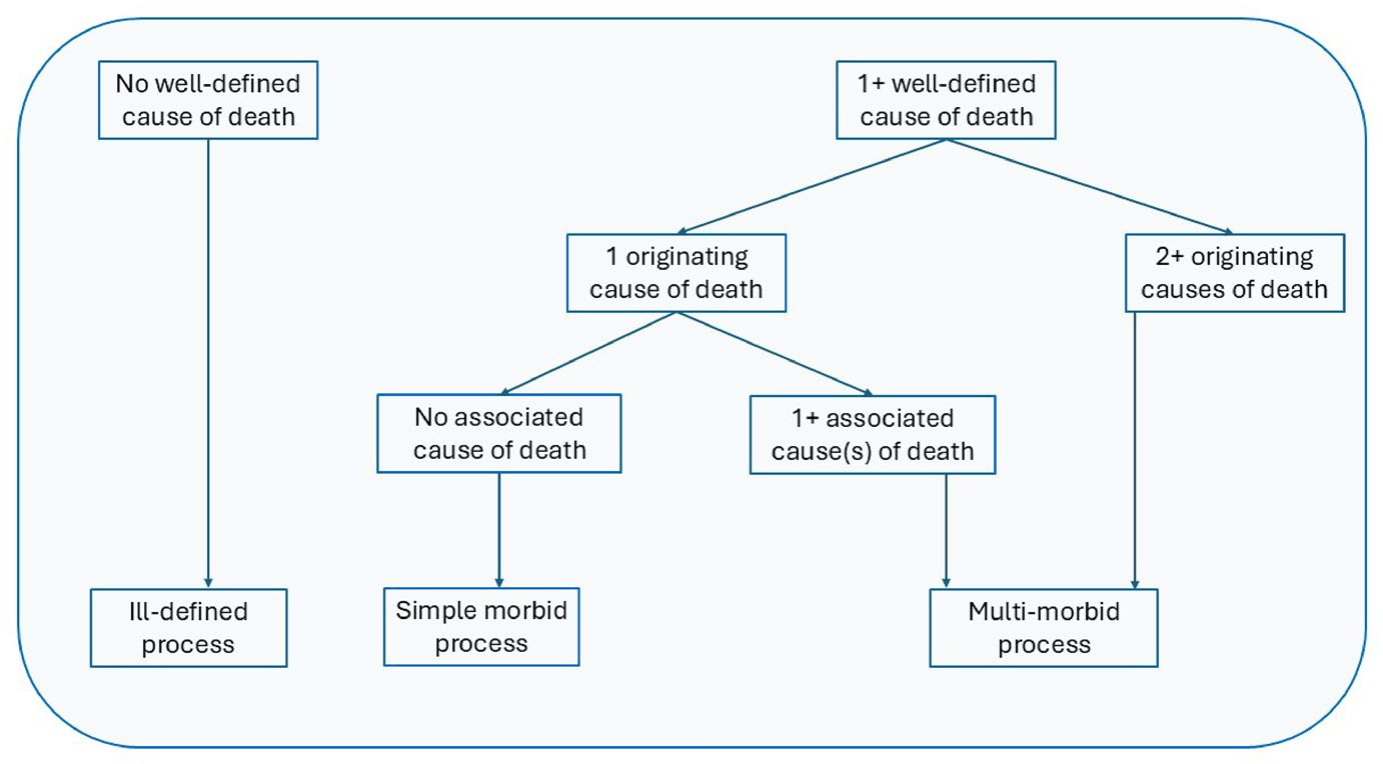
Classifying deaths according to the morbid process

Following an approach developed by Grippo et al., (2020, 2024), all deaths in our study are classified into one of three types of morbid processes: simple causal processes, multi-morbid processes, and ill-defined processes. Among all multiple causes of death, we identified the originating causes for each morbid process, that is the conditions that initiated the sequence of events that led directly to death, using the decision tables embedded in the IRIS system, a software implemented worldwide to automate coding of all causes of death reported on death certificates into ICD-10 and to identify the underlying cause of death (www.iris-institute.org). We define simple processes as those characterizing deaths with a single originating cause and no associated causes listed on Part 2 of the death certificate; multi-morbid processes as those characterizing deaths with associated causes or with more than one originating cause; and ill-defined processes as those characterizing deaths with only ill-defined causes, that is causes listed on the death certificate as unknown or as symptoms, signs or those that are poorly informative.^2^ Figure 1 summarizes the classification process and we refer the reader to Grippo and colleagues (2024) for additional details on the method.

From the tabulation of deaths in each country by five-year age group, sex, and morbid process, we calculated the corresponding age-specific death rates using exposures from the Human Mortality Database ( Barbieri, 2015; HMD, 2022) for each of the four countries. From the age-specific death rates, we calculated age-standardized death rates, using the US exposures from the HMD as the reference. The rates are used to determine the contribution of the three types of morbid process to the difference in life expectancy between the US and each of the three comparison countries using a decomposition method proposed by Pollard (Pollard, 1988).

We also estimated the total and average number of years of life lost by the US, overall and separately from each type of morbid process, adapting a method developed by Preston and Vierboom (2021). First, we estimated the distribution of excess deaths in the US by sex, age group, and morbid process. We did so by applying the age- and sex-specific death rates for the three morbid processes of each comparison country to the US population in turn, and taking the difference between the actual and the estimated death counts. We then multiplied excess deaths in each age group and for each type of morbid process by the life expectancy at the corresponding age in the 2017 lifetable for the three comparison countries separately for each sex. Adding up all those years provided an estimate of the total number of years of life lost to the US if all these excess deaths could have been averted and had then been subjected to the mortality rates of the comparison countries. Dividing the total number of years of life lost by the overall number of excess deaths provided an estimate of the average number of years lost per excess death.

## Results

We successively present the results of our analysis regarding differences in the age-standardized death rates, the age-specific death rates, the life expectancies, the excess death counts and the lost years of life, thus moving from the simplest to the most complex measures of mortality, each providing a different angle to the analysis. We describe the differences between the US and peer countries overall and for each type of morbid process, as well as by sex when the patterns differ for men and for women.

### Age-standardized death rates

At 870 per 100,000 population for both sexes and all morbid process combined, the age-standardized death rate in the US is 30–40 percent higher than in the comparison countries. Though rates are much higher for men, both the absolute and the relative differences are larger for women: from 28 percent higher than in Italy to 40 percent higher than in France and Spain, compared with about 20 percent higher for men relative to each of the three peer countries.

We looked at the distribution of deaths across the three types of morbid processes in the four study countries and found that, for men, the age-standardized death rate attributable to simple processes represents 52% of the overall rate in France, 48% in Italy, 60% in Spain, and 49% in the US. The corresponding figures for multi-morbid processes are 37, 48, 37, and 48%. For ill-defined processes, they are 11, 4, 3, and 3%. For women, the shares are 54, 51, 61 and 52% for simple processes in France, Italy, Spain, and the US, respectively; 34, 45, 35, and 44% for multi-morbid processes; and 12, 4, 4, and 4% for ill-defined processes. These values thus show a similar share of multi-morbidity in the US than in Italy and a higher share than in France and Spain, for both males and females (Table 1).

**Table 1:** Total number of deaths and life expectancy at birth by sex in France, Italy, Spain and the United States in 2017 and differences (in years) between each comparison country and the United States

Country	Sex	Total death count	Life expectancy at birth	Difference in life expectancy (peer-US)
France	Men	292,874	79.4	3.2
	Women	298,656	85.3	4.1
	Both sexes	591,530	82.4	3.7
Italy	Men	310,575	80.4	4.2
	Women	340,015	84.9	3.7
	Both sexes	650,590	82.7	4.0
Spain	Men	214,236	80.3	4.1
	Women	210,287	85.7	4.5
	Both sexes	424,523	83.0	4.3
The United States	Men	1,439,020	76.2	
	Women	1,374,354	81.2	
	Both sexes	2,813,374	78.7	

*Source*: Authors’ calculations using cause-of-death data from national vital statistics systems of France (CepiDc-INSERM), Italy (ISTAT), Spain (INE), and the United States (NCHS), and population data from the Human Mortality Database (HMD, 2022)

However, when we look at how each type of process contributes to the difference in the age-standardized death rate between the US and peer countries, we find that the contribution of multi-morbid processes is much larger than for simple processes when comparing the US with France or Spain: the difference reaches 165 per 100,000 in absolute value for both sexes combined in the two countries, *versus* 83 and 32 per 100,000, respectively, for simple morbid processes (Table 2). When comparing the US with Italy, simple morbid processes contribute a little bit more than multi-morbid processes to the difference (i.e., for 96 and 81 per 100,000 for simple and multi-morbid processes). The pattern is very similar for men and for women though, again, rates are smaller for the latter. Note that the US experiences relatively low mortality from ill-defined processes, especially when compared with France which has the highest share of deaths from ill-defined causes among all four study countries. The age-standardized death rate from ill-defined process for both sexes combined stood at 78 per 100,000 in France compared with 31 in the US, 28 in Italy, and 24 in Spain.

**Table 2:** Age-standardized death rates (all ages) per 100,000 by country, sex, and type of morbid process, 2017

Type of morbid process	Males				Females				Total			
	France	Italy	Spain	The US	France	Italy	Spain	The US	France	Italy	Spain	The US
Simple	457.3	414.9	519.8	510.7	278.7	284.8	315.9	377.5	353.9	340.2	404.7	436.6
Multi-morbid	324.2	417.1	314.6	506.4	173.4	255.3	181.5	319.7	235.8	320.9	236.8	401.8
Ill-defined	95.4	31.3	28.5	30.6	63.1	24.3	19.1	30.7	78.0	27.9	23.7	31.2
All	876.9	863.3	862.9	1047.7	515.2	564.5	516.5	727.9	667.7	688.9	665.3	869.6

*Source*: Authors’ calculations using cause-of-death data from national vital statistics systems of France (CepiDc-INSERM), Italy (ISTAT), Spain (INE), and the United States (NCHS), and population data from the Human Mortality Database (HMD, 2022)

### Age-specific death rates

**Fig. 2:**
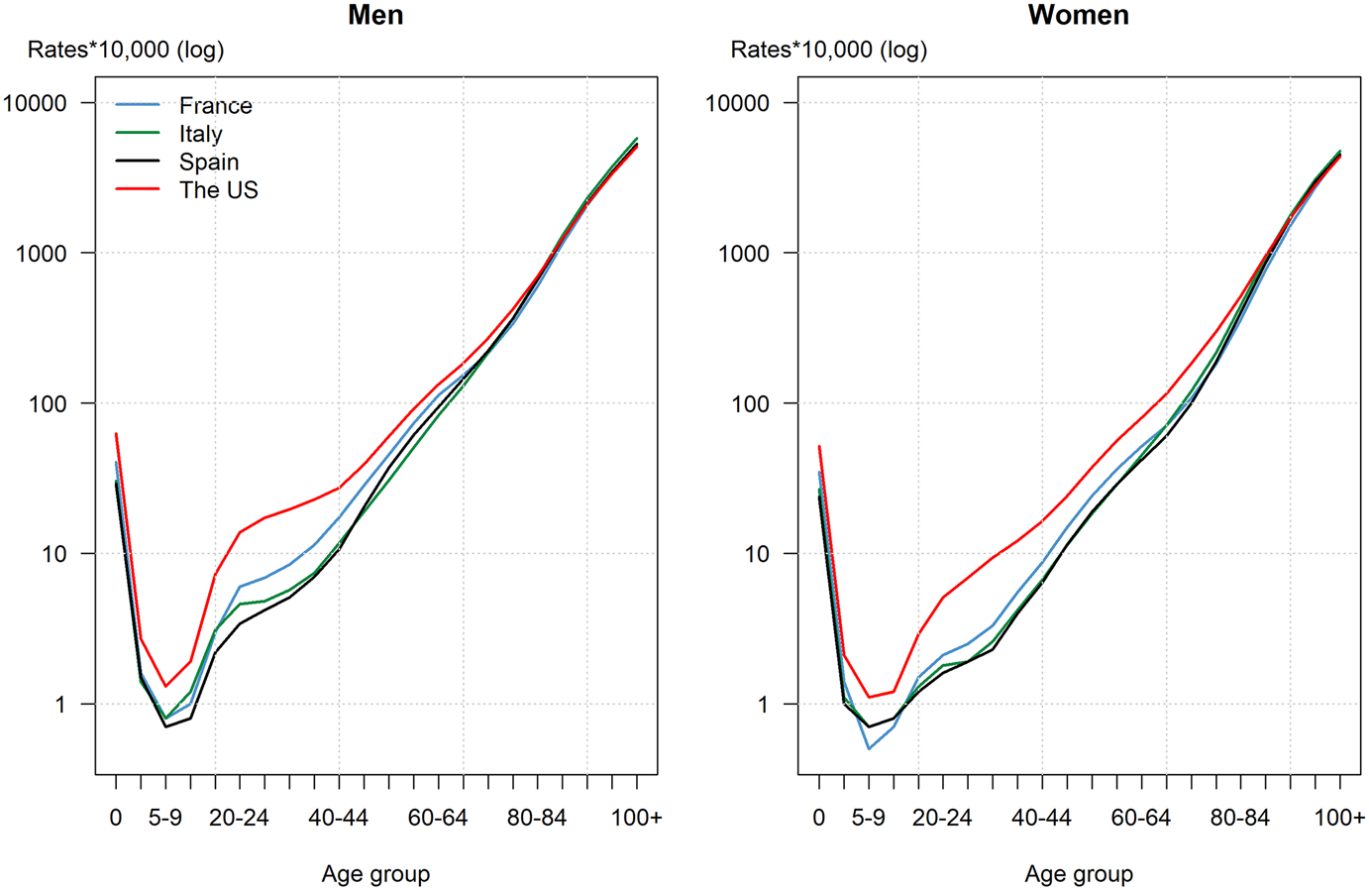
Age-specific mortality rates by sex in each country, all morbid processes combined. *Source*: Human Mortality Database (HMD, 2022)

The marked US disadvantage in mortality is not uniformly distributed across the age range (Appendix Tables 4-7). Mortality rates were higher in the US than in the three comparison countries for each sex and all ages up to 85 years for men and 95 years for women, but similar or very slightly lower at older ages (Fig. 2).

**Fig. 3:**
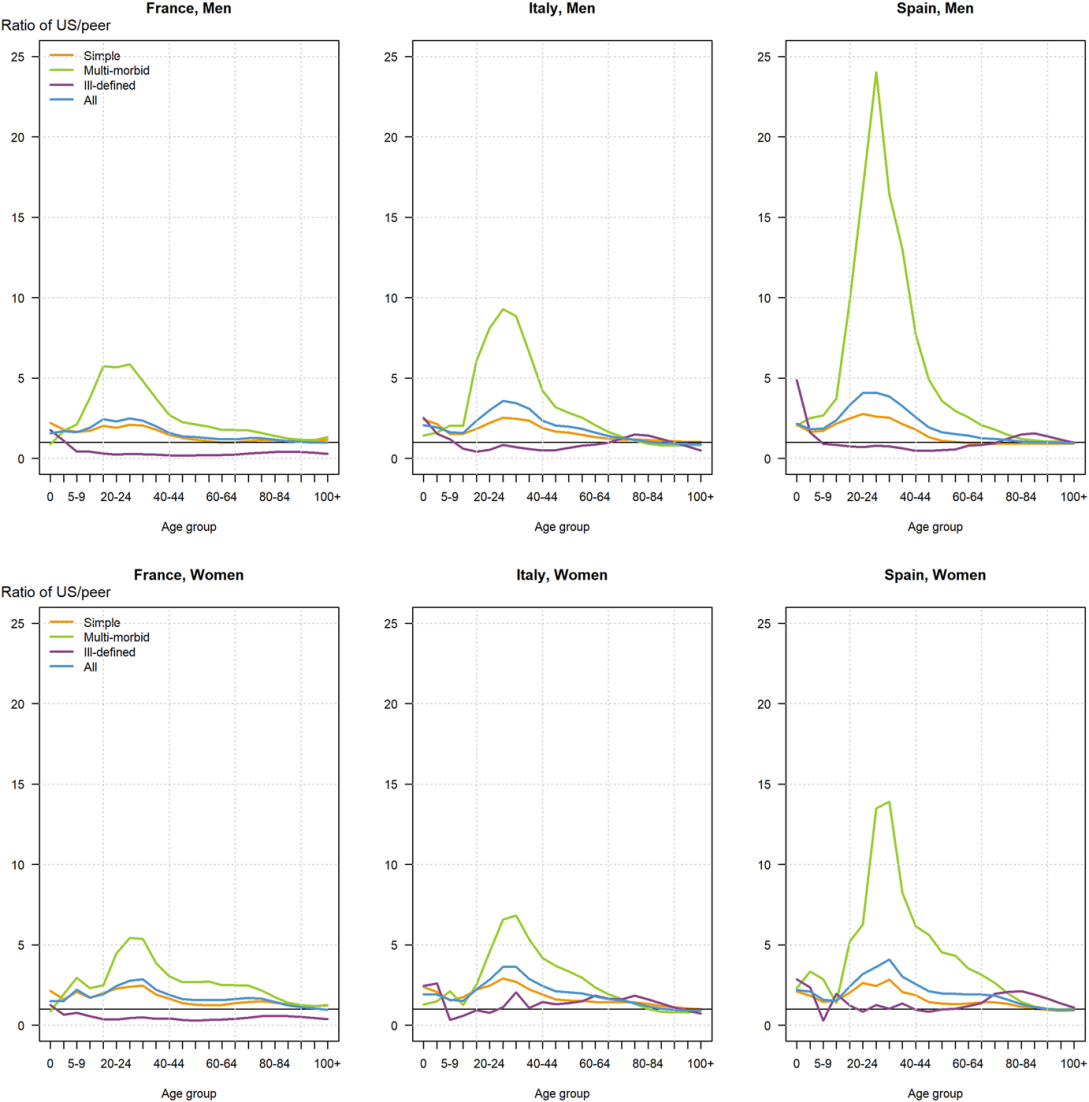
Age-specific mortality rate ratio of US compared to peer countries by morbid process, for each sex, 2017. *Source*: Authors’ calculations using cause-of-death data from national vital statistics systems of France (CepiDc-INSERM), Italy (ISTAT), Spain (INE), and the United States (NCHS), and population data from the Human Mortality Database (HMD, 2022)

More specifically, at 6.3 per 1,000 for boys and 5.2 per 1,000 for girls, infant mortality is high in the US compared with France (where these same figures reach 4.0 and 3.5 per 1,000) and even more so compared with Italy (where they stand at 3.1 and 2.7 per 1,000) and Spain (at 2.9 and 2.4 per 1,000). However, the US disadvantage is particularly pronounced at ages 15–40 years. The maximum is reached at 25–29 years, when rates are 2.5 times higher for males than in France, 3.6 times than in Italy, and 4.1 times than in Spain. For that same age group, the ratios for females reach 2.8, 3.6 and 3.6 (though the ratio is higher in the age group 30 to 34 years compared to Spain only, at 4.1). Death rates in the US are also much higher for the surrounding age-groups, that is at 15–29 and 34–39 years for both sexes, especially relatively to Italy and Spain. The US disadvantage disappears at higher ages, that is starting at age 85 years compared with Spain and Italy and 95 compared with France. The US even exhibits a slight advantage compared with Italy at ages 85 years and above for men, 90 years and above for women but far from enough to compensate for the disadvantage at younger ages.

Figure 3 represents the ratio of the US age-specific mortality rates for each of the three morbid processes (simple, multi-morbid, and ill-defined) as well as for all morbid processes combined, in relation to those in each of the three comparison countries, separately for each sex.

At ages 25–29 years, when excess mortality in the US reaches its peak, the age-specific death rate for men from multi-morbid processes is 79 per 100,000 compared with 3 in Spain and 14 in Italy and in France. Thus, the US rate is 5 times higher than in France and Italy and an astonishing 26 times higher than in Spain. The corresponding rate for simple morbid processes in the US is higher, at 90 per 100,000, but closer to that in the three other countries (i.e., 34 in Spain, 42 in France, and 30 in Italy), thus “only” 2–3 times higher. For women, these values follow the same pattern, with US mortality rates from multi-morbid processes also 5–6 times higher than in France and Italy, though the gap with Spain is not as large as for men, with a US rate 14 times higher.

Excess mortality from multi-morbid processes in the US is also marked for the surrounding age groups (20–24 for men and 30–34 years for both men and women), though not as pronounced. The ratios then decline progressively from these age groups to the two extremes of the age distribution. Compared to Italy only, they even reverse at ages 80 years and above for both sexes, when the age-specific mortality rates from multi-morbid processes are about 10% lower in the USA. Except for these higher ages and for children below the age of 5, excess US mortality from multi-morbid processes is systematically higher than excess mortality from simple morbid processes for which the ratios never reach above 2.1 in any age group compared with France, 2.8 compared with Spain, and 3 compared with Italy (Fig. 3).

It is also worth noting that the previously mentioned (and only) relative advantage exhibited by the US as regards mortality from ill-defined processes compared to France and, to a smaller extent, to Italy, is particularly marked at ages below 65 years. The prevalence of deaths from ill-defined causes reflects differing certification practices across countries and variations in the understanding of when it is legitimate to attribute death to an ill-defined cause. Such differences could bias our results since, in practice, selecting an ill-defined cause increases the chance that a death is taken out of the pool of single or multi-morbidity processes. We further discuss the potential impact of these variations on our findings in the limitation section of this article.

**Table 3:** Excess deaths, total number of person-years lost to excess mortality, and average number of years of life lost per excess deaths by sex and morbid process in the United States assuming survival rates of France, Italy, and Spain, 2017

Mortality indicator	France			Italy			Spain		
	Males	Females	Both sexes	Males	Females	Both sexes	Males	Females	Both sexes
*Excess US deaths from*									
- Simple processes	102 092	204 891	314 518	145 555	162 804	311 888	− 1 864	99 773	100 713
- Multi-morbid processes	235 469	230 755	473 918	147 996	93 359	247 943	291 307	237 366	531 708
- Ill-defined processes	− 84 861	− 61 332	− 145 477	− 712	10 767	9 870	1 056	22 894	23 849
- All processes	252 701	374 314	642 958	292 839	266 929	569 701	290 499	360 033	656 270
*Total years of life lost to all excess deaths from*									
- Simple processes	2 856 368	3 749 957	6 643 218	4 251 191	3 706 534	8 016 708	1 851 849	2 999 711	4 774 847
- Multi-morbid processes	5 403 383	4 540 235	10 100 078	5 310 649	3 678 028	9 130 861	7 216 969	5 502 292	12 888 816
- Ill-defined processes	− 1 515 750	− 728 042	− 2 297 250	− 66 679	185 336	102 745	− 89 611	224 029	116 270
- All processes	6 744 001	7 562 149	14 446 046	9 495 162	7 569 897	17 250 314	8 979 207	8 726 031	17 779 933
Average years of life lost per excess death from*									
- Simple processes	28.0	18.3	21.1	29.2	22.8	25.7	NA	30.1	47.4
- Multi-morbid processes	22.9	19.7	21.3	35.9	39.4	36.8	24.8	23.2	24.2
- Ill-defined processes	NA	NA	NA	NA	17.2	10.4	NA	9.8	4.9
- All processes	26.7	20.2	22.5	32.4	28.4	30.3	30.9	24.2	27.1

^*^ "NA" values are indicated where the United States had no excess deaths compared to peer

*Source*: Authors’ calculations using cause-of-death data from national vital statistics systems of France (CepiDc-INSERM), Italy (ISTAT), Spain (INE), and the United States (NCHS), and population data from the Human Mortality Database (HMD, 2022)

### Life expectancy at birth

Life expectancy at birth in 2017 was 78.7 years for both sexes combined in the US (76.2 years for men and 81.2 years for women), thus much lower than in the other three countries (82.4 in France, 82.7 years in Italy, and 83.0 years in Spain for both sexes combined) (Table 1). This means that the US is only now reaching the level of life expectancy at birth already attained by its peers around the turn of the century. The gap in life expectancy at birth with the US reached 3.7 years (compared with France), 4.0 (with Italy) and 4.3 (with Spain) years for both sexes combined, 3.2, 4.2, and 4.1 years for men, and 4.1, 3.6, and 4.5 years for women, respectively.

**Fig. 4:**
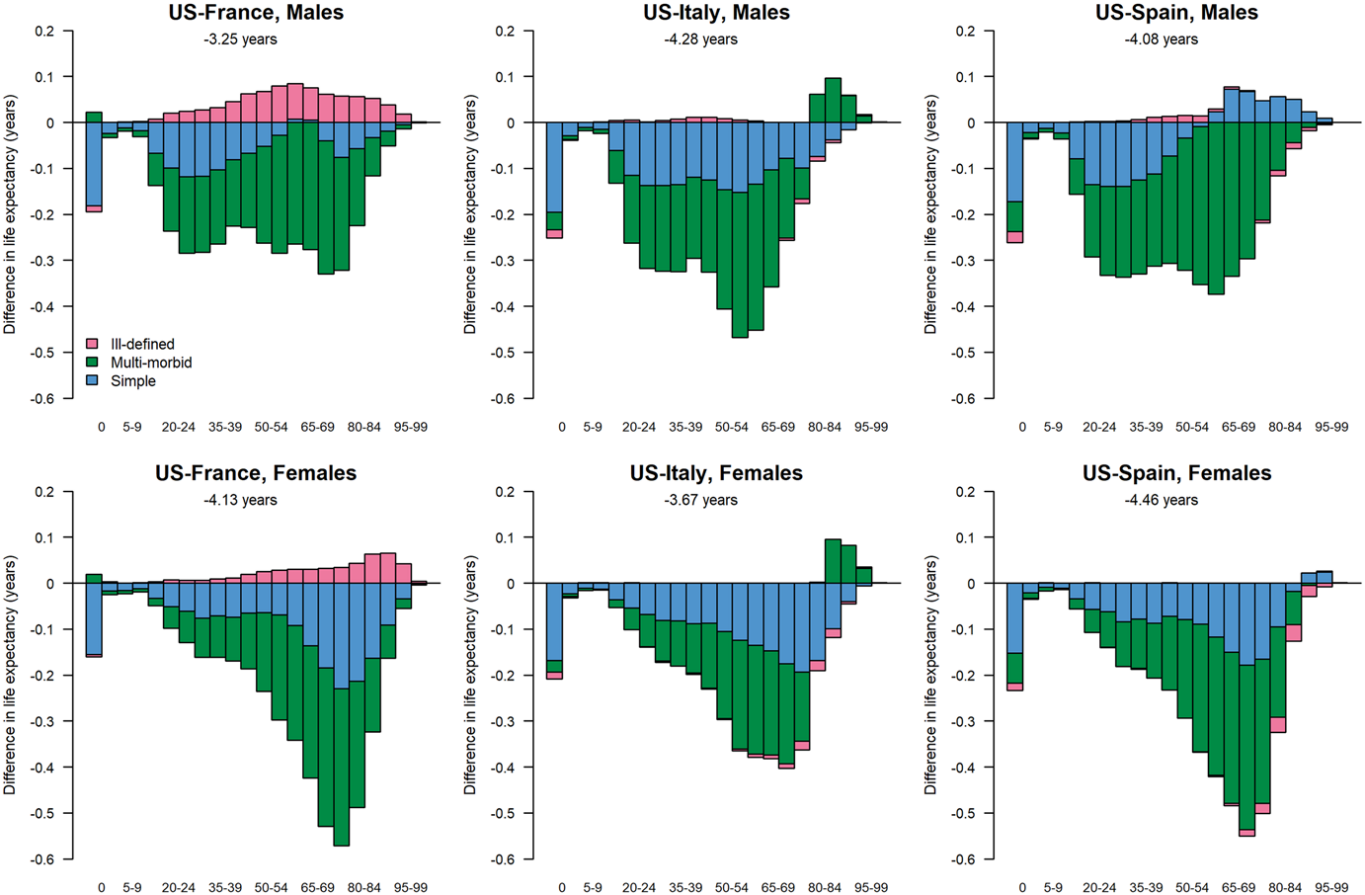
Contribution (in years) of the three morbid processes to the difference in life expectancy at birth between the US and peer countries, each sex. *Source*: Authors’ calculations using cause-of-death data from national vital statistics systems of France (CepiDc-INSERM), Italy (ISTAT), Spain (INE), and the United States (NCHS), and population data from the Human Mortality Database (HMD, 2022)

Overall, multi-morbid processes contributed 51% to the US gap in life expectancy with Italy, 75% with France, and 73% with Spain while simple morbid processes contributed 48, 42, and 26% for both sexes combined for each of the three comparison countries. The contribution of multi-morbid processes tends to be larger for men than for women (Appendix Tables 8 and 9). Our analysis indicates that if the mortality rates from multi-morbid processes had been the same in the US as in the comparison countries, the US would have experienced a smaller gap with other countries. The gap in life expectancy at birth would have been reduced by 2.4 years compared with Italy, 2.9 years with France, and 3.4 years with Spain for men. For women, the gaps would have been reduced slightly less, i.e., by 1.7, 2.7, and 2.8 years, respectively (Fig. 4).

The mortality burden of multi-morbid processes varies across the age range. Below age 20 years, the loss in life expectancy in the US from any kind of morbid processes is small, i.e., 0.3 to 0.4 years overall depending on the comparison country and sex, and nearly all of it is attributable to simple processes. This is related to the fact that, though mortality at younger ages, and especially during the first year of life, is more than twice as high in the US than in the comparison countries for simple morbid events, the gap is much smaller for mortality from multi-morbid processes.

The deficit in years lived is disproportionately large in the US for working-age adults compared with peer countries. The loss of years of life at ages 20 to 64 years reaches 1.8 years compared with France (1.9 years for men, 1.6 years for women), 2.5 years compared with Spain (2.9 and 2.1 years for men and women, respectively), and 2.6 years compared with Italy (3.1 and 2.1 years), representing 60 to 75% of the total loss in life expectancy for men and 40 to 55% for women. In this age group, most of the loss is attributable to multi-morbid processes, especially for men for whom this type of processes contributes for 63% in Italy, 77% in Spain and 88% in France. For women, the corresponding proportions reach 59, 66 and 71%, respectively (Appendix Tables 8 and 9).

Between the ages of 65 and 80 years, the US disadvantage is smaller than at younger ages but multi-morbid processes continue to play an overwhelming role in the loss of life years. For men, the US loss attributable to multi-morbid processes reaches 0.8 years compared with France and Spain and 0.5 years with Italy. By contrast, the loss attributable to simple processes is only 0.1 compared with France and 0.3 years with Spain. However, the US actually experiences an advantage compared with Italy, where mortality is higher for simple processes, with the US gaining 0.2 years of life from this type of morbid processes at these higher ages. For women, the US is at a disadvantage compared with all three peer countries and the loss of life is larger for both types of processes: for multi-morbid processes, the loss ranges from 0.6 years compared with Italy to a whole year compared with Spain; for simple processes, it reaches half a year with all three peers.

At age 80 years and above, as previously mentioned, the US disadvantage becomes much smaller with a life expectancy at age 80 years at 8.5 years for men and 9.8 years for women compared with 8.3 and 10.1 years in Italy, 8.6 and 10.5 years in Spain, and 8.9 and 11.0 years in France. The contribution of simple versus multi-morbid processes is much less consistent across countries than at younger ages. Compared with France, both simple and multi-morbid processes are disadvantageous to the US life expectancy for both men and women, though higher mortality from ill-defined processes partly compensates for the loss in years of life incurred by the other types of processes. In Italy, mortality from simple processes results in a very small loss in the US but it is largely compensated by the contribution of multi-morbid processes, explaining the longer life expectancy at age 80 years in the US. In Spain, we observe the reverse pattern, where the US experiences losses in years of life from multi-morbid processes but (minuscule) gains from simple processes.

### Excess deaths and lost years of life

Between one fifth and one quarter of all deaths would have been averted in the US if the country had experienced the 2017 mortality rates of Italy (20%), France (23%), or Spain (23%) (Table 3). In France and Spain, a very large proportion of these averted deaths would have been the result of multi-morbid processes. The share of deaths from multi-morbid process in the US was 49% for men and 44% for women, compared with 49% and 52% for deaths from simple processes. Excess US deaths from multi-morbid processes represented, respectively, 17 and 19% of the total number of deaths in the US compared with 11 and 4% for those resulting from simple morbid processes. The total number of deaths averted from simple and multi-morbid processes for the two sexes combined would have represented, respectively, 11 and 9% of all US deaths with the mortality rates of Italy, 11 and 17% with those of France, and 4 and 19% with those of Spain. Americans whose deaths would have been averted if they had experienced the mortality risks of their more advantaged peers could have been expected to live for an additional 23 years with the lifetable values of France (27 years for men and 20 years for women), 27 years with those of Spain (31 years for men and 24 years for women), and 30 years with those of Italy (32 years for men and 28 years for women). These large values reflect the fact that excess US deaths tend to be concentrated at relatively young adult ages, when the potential for additional years of life is high.

## Study limitations

We cannot rule out the fact that differences in certifying practices across the four study countries could influence some of the results. An important issue is the previously mentioned high share of deaths coded to ill-defined or unknown causes in France compared to the US, which could mask higher rates of mortality from simple and multi-morbid processes. To evaluate the impact of the differential rate of ill-defined causes on the cross-country comparison, we experimented with different scenarios for the redistributions of deaths from ill-defined and unknown causes for all four countries. We found that all these strategies made no material differences to the results of our analysis because the lower rate of mortality from ill-defined deaths in the US compared to France and Italy is far from sufficient to compensate for its higher rates from simple and multi-morbid processes.

Similarly, we noticed that in Spain, deaths requiring forensic investigations, which are in large part those due to accidents and are relatively more frequent at young ages, are automatically assigned a single cause of death.^3^ This practice could have resulted in the lower proportion of multi-morbid processes than in the other countries and, consequently, in the much higher ratio of age-specific death rates from this type of processes in the US compared to Spain. Nonetheless, it remains that even in France and Italy, where there is no such practice, the disadvantageous pattern observed in the US as regards multi-morbidity is very similar than in Spain, though more muted for young working-age adults than is found in this latter country.

Unfortunately, we lack additional information to assess the comparability of the multiple cause-of-death data across the four study countries, such as the autopsy rate, the share of certificates that have been filed electronically, or the percentage of deaths for which information on the medical cause of death was provided by a non-physician. Our findings must thus be taken with a grain of salt. However, the plausibility of our results considering the literature on the US disadvantage in health and mortality and the high degree of consistency in the pattern of differences between the US and each of the three peer countries give us reasonable confidence that our overall results are largely grounded on reality.

## Discussion

Our results indicate that multi-morbid processes contribute overwhelmingly to the large gap in life expectancy between the US and the three comparison countries, France, Italy, and Spain, for both men and women (though slightly muted for the latter relative to the former). They also show that the disadvantage in the mortality rates from multi-morbid processes operates throughout most of the age range, though it is particularly concentrated among working-age adults. The pattern is very similar across all countries below the age of 85 years, above which the US experience a lower level of mortality from multi-morbid processes compared with Italy only. Within the most disadvantaged age range (20 to 64 years), the gap in mortality rates is largest at 25-29 years while the contribution of multi-morbid processes increases progressively and reaches a peak at ages 55 to 64 years when the share of multi-morbidity to the difference in life expectancy at birth between the US and peers is most pronounced. These results are consistent with the literature, which shows that the US disadvantage in mortality is exceptionally large for working age adults in general, and for young adults in particular (Harris et al., 2021; Ho, 2013; Ho & Preston, 2010). The difference in age pattern in the contributions to life expectancy and in the ratio of the mortality rates is due to the fact that large variations in small rates contribute less to gaps in life expectancy at birth than small variations in large rates. The result remains that all age groups between 20 and 85 years contribute significantly to the US disadvantage in the length of life for men: for young working-age adults this is because the relative differences in rates are large and also because a death averted at young ages results in more years of life expectancy gained than a death averted at older ages, while for older adults, the contribution is due to large absolute differences in the rates.

Because each disease comes with its own risk factors, the disproportionate prevalence of multi-morbidity at death in the US compared with peer countries suggests that no single factor is likely to explain the difference and a broad range of determinants is probably at play in the country excess mortality and lag in life expectancy. An in-depth analysis by the National Research Council conducted in the early 2010s explained the US shortfall in life expectancy as multi-factorial: differences in health systems, individual behaviors, the distribution and impact of education and income on mortality, economic inequalities, the environment, and policies or the lack thereof, in particular as regards social welfare and income redistribution programs, as well as social values and norms all appear to contribute (National Research Council, 2013). Particularly relevant for our findings, this study and other more recent ones have shown that all these determinants operate through proximate factors like higher rates of obesity, hypertension, diabetes, and substance abuse, often identified on death certificates as associated or contributing causes of death (Woolf, 2023). These conditions are best controlled through prevention but a report by the National Academies of Sciences, Engineering, and Medicine found that the primary care system is failing in the US (2021). A recent publication by the Commonwealth Fund which included a range of high-income countries notes that the US ranks last on nearly all indicators of health care system performance (Schneider et al., 2021). According to this study, what more specifically appears to distinguish the top-achieving countries from the US is that "1) they provide for universal coverage and remove cost barriers; 2) they invest in primary care systems to ensure that high-value services are equitably available in all communities to all people; 3) they reduce administrative burdens that divert time, efforts, and spending from health improvement efforts; and 4) they invest in social services, especially for children and working-age adults" (Schneider et al., 2021, p. 2). It is thus unlikely that a unique intervention, program, or policy will be sufficient to close the gap between the US and its peers. In the meantime, the disproportionate burden of multi-morbidity in the US will continue to complicate efforts to sustain progress in life expectancy in this country and to improve health and the quality of life of the general population, and it is very likely to put additional pressure on the already extraordinary cost of health care compared to peers (Papanicolas, Woskie, Jha, 2018).

Indeed, the simultaneous presence of at least two independent diseases or conditions in a single individual creates multiple challenges for the health care system. Fortin and colleagues emphasize how “multi-morbidity affects processes of care and may result in complex self-care needs; challenging organizational problems (accessibility, coordination, consultation time); polypharmacy; increased use of emergency facilities; difficulty in applying guidelines; and fragmented, costly, and ineffective care”, thus leading to “decreased quality of life, psychological distress, longer hospital stays, more postoperative complications, and a higher cost of care” (Fortin et al., 2007, p.1016). More recent studies have confirmed that multi-morbidity is associated with increased mortality risks, disability, reduced functional status, poor quality of life, complicated treatment options, and adverse drug events due to incompatibilities, calling for coordinated and comprehensive care (Brettschneider et al., 2013; Calderon-Larranaga et al., 2012; Chowdhury et al., 2023; Forman et al., 2018; Wolff et al., 2005). Multi-morbidity is associated with more health care use and longer hospitalizations (Ofosi-Asenso et al., 2019). Multi-morbidity also poses particular challenges to the health care system which, in most countries, is still designed to manage a single disease with a highly segmented system of care (Barnett et al., 2012; DuGoff et al., 2014; Marengoni et al., 2011; Wallace et al., 2015).

Our study has confirmed that an approach based on the multiple cause-of-death information available on death certificates is a very valuable complement to the analysis of multi-morbidity in living populations, especially for international comparisons. The approach we followed overcomes many of the shortcomings from observational studies or from survey data because it relies on routinely collected vital statistics based on a standard data collection instrument (the WHO recommended death certificate with all causes of death coded to the International Classification of Diseases) and unified definitions. It therefore provides widespread, systematic, low cost, and highly comparable information on multi-morbidity at death. In addition to international comparisons, this approach is easily amenable to additional studies of changes in the prevalence of multi-morbidity at death over time or within-population variations by geographic location, race/ethnicity, or social group. Death certificates are not a substitute to survey data because the type of multi-morbidity reported does not neatly overlap with the multi-morbidity measured with survey data or in observational studies: certifiers are indeed only expected to report those causes that might have contributed either directly or indirectly to the death while, in living populations, multi-morbidity is typically defined as the co-occurrence of two or more chronic conditions, including some which can reduce quality of life but do not necessarily have life threatening consequences. Our approach should thus be considered as a useful complement, rather than as an alternative, to the measurement of multi-morbidity in living populations.

## Data Availability

Available on demand.

## Appendix

See Tables 4, 5, 6, 7, 8 and 9.

**Table 4:** Age-specific and age-standardized (ASDR) mortality rates from all morbid processes combined by country and by sex (p. 100,000), 2017

Age	Males				Females				Total			
group	France	Italy	Spain	The US	France	Italy	Spain	The US	France	Italy	Spain	The US
0	405	305	292	627	349	268	239	519	378	287	266	574
1–4	16	14	15	27	14	11	10	21	15	13	13	24
5–9	8	8	7	13	5	7	7	11	7	8	7	12
10–14	10	12	8	19	7	8	8	12	8	10	8	16
15–19	30	31	22	73	15	13	12	29	23	22	17	51
20–24	60	46	34	138	21	18	16	51	40	33	25	96
25–29	69	48	42	172	25	19	19	69	46	33	30	121
30–34	84	57	51	196	33	26	23	94	58	42	37	145
35–39	114	74	70	228	55	42	40	121	84	58	55	175
40–44	174	117	107	274	87	67	64	164	130	92	86	219
45–49	283	190	203	390	150	114	115	242	216	152	159	315
50–54	456	306	373	603	241	184	189	375	347	244	281	487
55–59	735	505	615	922	363	288	290	565	543	393	450	739
60–64	1132	832	948	1338	517	451	419	801	810	635	676	1058
65–69	1538	1310	1456	1838	709	714	606	1160	1101	998	1011	1479
70–74	2155	2182	2240	2713	1083	1209	1001	1839	1579	1662	1573	2243
75–79	3388	3668	3684	4233	1829	2176	1898	3000	2515	2838	2682	3549
80–84	6002	6821	6609	6954	3584	4451	3990	5157	4549	5416	5047	5912
85–89	11,499	13,031	12,233	12,321	7694	9338	8596	9509	9000	10,630	9894	10,578
90–94	20,717	23,126	21,444	21,214	15,236	17,828	16,891	17,022	16,729	19,303	18,241	18,353
95–99	34,302	37,348	34,518	33,535	27,245	30,840	29,671	28,152	28,656	32,215	30,789	29,471
100 +	52,409	57,642	53,165	50,815	45,662	47,704	44,997	43,859	46,493	49,198	46,322	45,000
ASDR*	877	863	863	1048	515	564	517	728	668	689	665	870

*Source*: Authors’ calculations using cause-of-death data from national vital statistics systems of France (CepiDc-INSERM), Italy (ISTAT), Spain (INE), and the United States (NCHS), and population data from the Human Mortality Database (HMD, 2022)

**Table 5:** Age-specific and age-standardized (ASDR) mortality rates from simple processes by country and by sex (p. 100,000), 2017

Age	Males				Females				Total			
group	France	Italy	Spain	The US	France	Italy	Spain	The US	France	Italy	Spain	The US
0	193	176	205	426	163	148	168	350	179	162	187	389
1–4	10	9	11	18	9	7	8	14	10	8	10	16
5–9	5	6	5	9	4	5	5	8	5	6	5	8
10–14	8	9	6	13	5	5	5	8	6	7	6	11
15–19	22	24	18	44	10	9	10	20	16	17	14	32
20–24	40	35	27	76	13	12	11	30	26	24	19	54
25–29	43	35	34	89	16	13	15	37	28	24	24	64
30–34	49	41	40	101	21	19	18	51	35	30	29	76
35–39	64	49	54	115	34	30	32	65	49	39	43	90
40–44	96	75	79	141	55	48	49	91	75	62	64	116
45–49	153	117	149	196	94	81	90	130	123	99	120	162
50–54	252	183	267	292	154	127	144	194	202	155	205	242
55–59	408	296	426	434	232	190	218	284	317	241	320	357
60–64	628	468	646	619	321	279	298	404	467	371	467	507
65–69	851	691	951	843	429	412	412	584	628	545	669	706
70–74	1165	1087	1389	1248	632	637	641	912	878	847	986	1067
75–79	1758	1684	2155	2002	1007	1068	1144	1507	1338	1341	1588	1728
80–84	3048	2940	3695	3370	1898	2020	2307	2651	2357	2395	2867	2953
85–89	5662	5578	6737	6082	3829	4202	4833	4962	4458	4683	5512	5388
90–94	10,088	10,127	11,779	10,821	7375	8189	9494	9046	8114	8729	10,171	9610
95–99	16,577	17,271	19,533	17,568	12,696	14,624	17,164	15,154	13,479	15,179	17,707	15,743
100 +	24,156	26,698	30,311	27,870	19,455	24,064	25,873	23,933	20,035	24,460	26,593	24,579
ASDR*	457	415	520	511	279	285	316	378	354	340	405	437

S*ource*: Authors’ calculations using cause-of-death data from national vital statistics systems of France (CepiDc-INSERM), Italy (ISTAT), Spain (INE), and the United States (NCHS), and population data from the Human Mortality Database (HMD, 2022)

**Table 6:** Age-specific and age-standardized (ASDR) mortality rates from multi-morbid processes by country and by sex (p. 100,000), 2017

Age	Males				Females				Total			
group	France	Italy	Spain	The US	France	Italy	Spain	The US	France	Italy	Spain	The US
0	189	113	79	162	161	108	61	139	176	111	70	150
1–4	4	4	3	7	3	4	2	5	3	4	2	6
5–9	2	2	1	4	1	1	1	3	1	2	1	3
10–14	1	3	1	5	1	3	2	3	1	3	2	4
15–19	5	5	3	28	3	3	2	8	4	4	2	18
20–24	11	7	4	60	4	4	3	20	7	6	3	41
25–29	14	9	3	79	5	5	2	30	9	6	3	55
30–34	19	10	6	91	8	6	3	41	13	8	4	66
35–39	29	16	8	108	14	10	6	53	21	13	7	81
40–44	47	30	17	128	23	17	11	69	35	23	14	98
45–49	83	59	38	187	40	29	19	107	62	44	29	146
50–54	141	105	84	300	65	52	39	174	103	78	61	236
55–59	238	187	160	472	100	92	63	271	167	138	110	369
60–64	393	336	271	695	154	164	109	382	268	247	188	532
65–69	548	584	464	961	223	289	177	553	377	430	314	745
70–74	808	1050	793	1410	359	545	338	884	567	780	548	1127
75–79	1346	1915	1446	2129	650	1056	708	1397	956	1437	1032	1723
80–84	2425	3733	2773	3376	1309	2291	1577	2283	1754	2878	2060	2742
85–89	4713	7069	5195	5773	2884	4735	3478	4003	3511	5551	4091	4676
90–94	8096	11,951	8932	9384	5420	8499	6644	6736	6149	9460	7323	7577
95–99	12,224	17,450	13,335	14,033	8935	13,452	10,647	10,492	9605	14,306	11,274	11,368
100 +	14,657	23,587	19,152	19,300	11,746	16,362	14,312	14,711	12,105	17,448	15,098	15,463
ASDR*	324	417	315	506	173	255	182	320	236	321	237	402

*Source*: Authors’ calculations using cause-of-death data from national vital statistics systems of France (CepiDc-INSERM), Italy (ISTAT), Spain (INE), and the United States (NCHS), and population data from the Human Mortality Database (HMD, 2022)

**Table 7:** Age-specific and age-standardized (ASDR) mortality rates from ill-defined processes by country and by sex (p. 100,000), 2017

Age	Males				Females				Total			
group	France	Italy	Spain	The US	France	Italy	Spain	The US	France	Italy	Spain	The US
0	22	15	8	39	24	12	11	30	23	14	9	35
1–4	2	1	1	2	2	1	1	2	2	1	1	2
5–9	1	0	0	0	0	0	1	0	1	0	0	0
10–14	1	1	0	0	1	1	0	0	1	1	0	0
15–19	3	3	1	1	2	1	0	1	3	2	1	1
20–24	9	4	3	2	3	1	1	1	6	3	2	2
25–29	13	4	4	3	4	2	1	2	8	3	3	3
30–34	16	6	6	4	5	1	2	2	10	4	4	3
35–39	21	8	8	5	7	3	2	3	14	5	5	4
40–44	31	11	11	5	9	3	4	4	20	7	8	5
45–49	47	14	15	7	15	4	6	5	31	9	11	6
50–54	62	17	22	11	22	5	6	6	42	11	14	9
55–59	89	22	30	17	31	7	10	10	60	14	20	13
60–64	111	28	31	24	42	8	12	14	75	17	21	19
65–69	139	34	40	34	58	14	17	23	96	24	28	28
70–74	182	44	57	55	92	27	22	43	134	35	38	49
75–79	284	69	83	102	172	52	47	96	221	60	62	99
80–84	529	148	141	208	378	140	106	223	438	143	120	217
85–89	1125	384	301	466	982	401	285	544	1031	395	291	514
90–94	2533	1048	734	1010	2441	1140	754	1239	2466	1114	747	1166
95–99	5501	2627	1650	1934	5614	2765	1860	2506	5571	2729	1808	2360
100 +	13,597	7358	3702	3645	14,461	7278	4812	5215	14,354	7290	4631	4958
ASDR*	95	31	28	31	63	24	19	31	78	28	24	31

*Source*: Authors’ calculations using cause-of-death data from national vital statistics systems of France (CepiDc-INSERM), Italy (ISTAT), Spain (INE), and the United States (NCHS), and population data from the Human Mortality Database (HMD, 2022)

**Table 8:** Contributions (in years) of type of morbid process to the difference in life expectancy at birth between each peer country and the United States for men by age group, 2017

Age group	US-France				US-Italy				US-Spain			
	Simple processes	Multi-morbid processes	Ill-defined processes	All processes	Simple processes	Multi-morbid processes	Ill-defined processes	All processes	Simple processes	Multi-morbid processes	Ill-defined processes	All processes
0	− 0.18	0.02	− 0.01	− 0.17	− 0.20	− 0.04	− 0.02	− 0.25	− 0.17	− 0.06	− 0.02	− 0.26
1–4	− 0.02	− 0.01	0.00	− 0.03	− 0.03	− 0.01	0.00	− 0.04	− 0.02	− 0.01	0.00	− 0.04
5–9	− 0.01	− 0.01	0.00	− 0.02	− 0.01	− 0.01	0.00	− 0.02	− 0.01	− 0.01	0.00	− 0.02
10–14	− 0.02	− 0.01	0.00	− 0.03	− 0.01	− 0.01	0.00	− 0.02	− 0.02	− 0.01	0.00	− 0.04
15–19	− 0.07	− 0.07	0.01	− 0.13	− 0.06	− 0.07	0.00	− 0.13	− 0.08	− 0.08	0.00	− 0.16
20–24	− 0.10	− 0.14	0.02	− 0.22	− 0.12	− 0.15	0.01	− 0.26	− 0.14	− 0.16	0.00	− 0.29
25–29	− 0.12	− 0.17	0.02	− 0.26	− 0.14	− 0.18	0.00	− 0.32	− 0.14	− 0.19	0.00	− 0.33
30–34	− 0.12	− 0.17	0.03	− 0.26	− 0.14	− 0.19	0.00	− 0.32	− 0.14	− 0.20	0.00	− 0.33
35–39	− 0.10	− 0.16	0.03	− 0.23	− 0.14	− 0.19	0.01	− 0.32	− 0.12	− 0.21	0.01	− 0.32
40–44	− 0.08	− 0.14	0.05	− 0.18	− 0.12	− 0.18	0.01	− 0.29	− 0.11	− 0.20	0.01	− 0.30
45–49	− 0.07	− 0.16	0.06	− 0.17	− 0.12	− 0.20	0.01	− 0.32	− 0.07	− 0.23	0.01	− 0.29
50–54	− 0.05	− 0.21	0.07	− 0.19	− 0.15	− 0.26	0.01	− 0.40	− 0.03	− 0.29	0.01	− 0.31
55–59	− 0.03	− 0.26	0.08	− 0.20	− 0.15	− 0.32	0.01	− 0.46	− 0.01	− 0.34	0.01	− 0.34
60–64	0.01	− 0.26	0.08	− 0.18	− 0.13	− 0.32	0.00	− 0.45	0.02	− 0.37	0.01	− 0.34
65–69	0.01	− 0.28	0.07	− 0.20	− 0.10	− 0.26	0.00	− 0.36	0.07	− 0.33	0.00	− 0.26
70–74	− 0.04	− 0.29	0.06	− 0.27	− 0.08	− 0.17	0.00	− 0.26	0.07	− 0.30	0.00	− 0.23
75–79	− 0.08	− 0.25	0.06	− 0.26	− 0.10	− 0.07	− 0.01	− 0.18	0.05	− 0.21	− 0.01	− 0.17
80–84	− 0.06	− 0.17	0.06	− 0.17	− 0.07	0.06	− 0.01	− 0.02	0.06	− 0.10	− 0.01	− 0.06
85–89	− 0.03	− 0.08	0.05	− 0.06	− 0.04	0.10	− 0.01	0.05	0.05	− 0.04	− 0.01	− 0.01
90–94	− 0.02	− 0.03	0.04	− 0.01	− 0.02	0.06	0.00	0.04	0.02	− 0.01	− 0.01	0.01
95–99	− 0.01	− 0.01	0.02	0.00	0.00	0.01	0.00	0.02	0.01	0.00	0.00	0.00
100 +	0.00	0.00	0.00	0.00	0.00	0.00	0.00	0.00	0.00	0.00	0.00	0.00
All ages	− 1.19	− 2.85	0.79	− 3.25	− 1.92	− 2.37	0.01	− 4.28	− 0.73	− 3.38	0.02	− 4.09

*Source*: Authors’ calculations using cause-of-death data from national vital statistics systems of France (CepiDc-INSERM), Italy (ISTAT), Spain (INE), and the United States (NCHS), and population data from the Human Mortality Database (HMD, 2022)

**Table 9:** Contributions (in years) of type of morbid process to the difference in life expectancy at birth between each peer country and the United States for women by age group, 2017

Age group	US-France				US-Italy				US-Spain			
	Simple processes	Multi-morbid processes	Ill-defined processes	All processes	Simple processes	Multi-morbid processes	Ill-defined processes	All processes	Simple processes	Multi-morbid processes	Ill-defined processes	All processes
0	− 0.16	0.02	0.00	− 0.14	− 0.17	− 0.03	− 0.01	− 0.21	− 0.15	− 0.07	− 0.02	− 0.23
1–4	− 0.02	− 0.01	0.00	− 0.02	− 0.02	− 0.01	0.00	− 0.03	− 0.02	− 0.01	0.00	− 0.04
5–9	− 0.02	− 0.01	0.00	− 0.02	− 0.01	− 0.01	0.00	− 0.02	− 0.01	− 0.01	0.00	− 0.02
10–14	− 0.01	− 0.01	0.00	− 0.02	− 0.01	0.00	0.00	− 0.01	− 0.01	0.00	0.00	− 0.01
15–19	− 0.03	− 0.02	0.00	− 0.05	− 0.04	− 0.02	0.00	− 0.05	− 0.03	− 0.02	0.00	− 0.06
20–24	− 0.05	− 0.05	0.01	− 0.09	− 0.05	− 0.05	0.00	− 0.10	− 0.06	− 0.05	0.00	− 0.11
25–29	− 0.06	− 0.07	0.01	− 0.12	− 0.07	− 0.07	0.00	− 0.14	− 0.06	− 0.08	0.00	− 0.14
30–34	− 0.08	− 0.09	0.01	− 0.16	− 0.08	− 0.09	0.00	− 0.17	− 0.08	− 0.10	0.00	− 0.18
35–39	− 0.07	− 0.09	0.01	− 0.15	− 0.08	− 0.10	0.00	− 0.18	− 0.08	− 0.11	0.00	− 0.19
40–44	− 0.07	− 0.10	0.01	− 0.16	− 0.09	− 0.11	0.00	− 0.20	− 0.09	− 0.12	0.00	− 0.21
45–49	− 0.06	− 0.12	0.02	− 0.17	− 0.09	− 0.14	0.00	− 0.23	− 0.07	− 0.16	0.00	− 0.23
50–54	− 0.06	− 0.17	0.03	− 0.21	− 0.10	− 0.19	0.00	− 0.30	− 0.08	− 0.21	0.00	− 0.29
55–59	− 0.07	− 0.23	0.03	− 0.27	− 0.12	− 0.24	0.00	− 0.37	− 0.09	− 0.28	0.00	− 0.37
60–64	− 0.09	− 0.25	0.03	− 0.31	− 0.14	− 0.24	− 0.01	− 0.38	− 0.12	− 0.30	0.00	− 0.42
65–69	− 0.14	− 0.29	0.03	− 0.39	− 0.15	− 0.23	− 0.01	− 0.38	− 0.15	− 0.33	− 0.01	− 0.49
70–74	− 0.18	− 0.35	0.03	− 0.50	− 0.18	− 0.22	− 0.01	− 0.40	− 0.18	− 0.36	− 0.01	− 0.55
75–79	− 0.23	− 0.34	0.03	− 0.54	− 0.19	− 0.15	− 0.02	− 0.36	− 0.17	− 0.31	− 0.02	− 0.50
80–84	− 0.21	− 0.28	0.04	− 0.45	− 0.17	0.00	− 0.02	− 0.19	− 0.10	− 0.20	− 0.03	− 0.32
85–89	− 0.16	− 0.16	0.06	− 0.26	− 0.10	0.10	− 0.02	− 0.02	− 0.02	− 0.07	− 0.04	− 0.13
90–94	− 0.09	− 0.07	0.07	− 0.10	− 0.04	0.08	0.00	0.04	0.02	0.00	− 0.02	− 0.01
95–99	− 0.03	− 0.02	0.04	− 0.01	− 0.01	0.03	0.00	0.03	0.02	0.00	− 0.01	0.02
100 +	0.00	0.00	0.00	0.00	0.00	0.00	0.00	0.00	0.00	0.00	0.00	0.00
All ages	− 1.91	− 2.69	0.46	− 4.14	− 1.91	− 1.65	− 0.12	− 3.68	− 1.51	− 2.79	− 0.16	− 4.47

*Source*: Authors’ calculations using cause-of-death data from national vital statistics systems of France (CepiDc-INSERM), Italy (ISTAT), Spain (INE), and the United States (NCHS), and population data from the Human Mortality Database (HMD, 2022)

## Footnotes

1 For Italy and Spain all deaths that occurred in the country are considered. For France deaths of people residing in overseas departments or abroad were not included in the data. For the US, only deaths of national and legal residents were included.

2 Here is the list of all ICD-10 codes corresponding to unknown or ill-defined causes of death as defined in our study: I46, I49.0, I95.9, I99, J96.0, P28.5, R00-R99.

3 For more information about this issue, see a note from the Spanish National Commission on Judicial Statistics here: https://datos.justicia.es/defunciones-con-intervencion-judicial [accessed December 6, 2024].
